# Endoscopic surgical treatment of bilateral coronal craniosynostosis

**DOI:** 10.3171/2021.1.FOCVID20136

**Published:** 2021-04-01

**Authors:** Yasser Jeelani, Mark R. Proctor

**Affiliations:** Department of Neurosurgery, Boston Children's Hospital, Harvard Medical School, Boston, Massachusetts

**Keywords:** bilateral coronal craniosynostosis, endoscopy, minimally invasive surgery

## Abstract

Endoscopic surgery for single-suture synostosis has been widely adopted since its introduction over 2 decades ago. Its role in syndromic synostosis is emerging, both as a primary treatment and as the first stage in a multimodal treatment paradigm aimed at preventing the vexing turribrachycephaly seen in these children. In this video, the authors review the technique for endoscopic treatment of bilateral coronal craniosynostosis and discuss both the benefits and some of the concerns to look out for over time. They also review the long-term outcomes in a consecutive series of patients treated in this fashion.

The video can be found here: https://vimeo.com/516351348.

## Transcript

This video will review the technique, indications, and outcomes for the endoscopic surgical treatment of bilateral coronal craniosynostosis.

**0:36 Modern Craniofacial Center**. Children with syndromic craniosynostosis can be quite complicated. They should ideally be treated in a multidisciplinary clinic where the option for both minimally invasive procedures and open cranial vault procedures are available.

**0:50 Recent Review of 500 Consecutive Patients**. In a recent review from our institution of 500 consecutive endoscopic cases, 34 cases, or approximately 7%, represented syndromic cases. Half of the cases were Apert syndrome. This will serve as the basis of the results in this talk.

**1:05 Rationale for Early Treatment in Bilateral Coronal Craniosynostosis**. The rationale for early treatment in bilateral coronal synostosis is to prevent the development of turribrachycephaly in the first year of life. Many centers use early posterior distraction to move out the back, even if the lambdoid sutures are open, as a way of preventing turricephaly. We have elected to release the fused coronal sutures to prevent the turricephaly, allow for frontal expansion, and ideally avoid a second and third operation, which is inherent in the distraction techniques.

**1:35 Surgical Video**. The patient is prepared for surgery with general anesthesia and two IVs. No arterial line is used. Hair is shaved in the appropriate area and two 2-cm incisions are used. It is important to obtain bone removal as far inferior as the lateral canthus and all the way to midline. A single dose of preoperative antibiotics are administered. After sterile prep and drape, the incisions are infused with local anesthesia. We then open using a Colorado needle to do this as bloodlessly as possible. Once the incisions are made, the underlying bone is identified and we attempt to place a burr hole directly where the suture would have been. Often, this is identifiable from a ridge, but occasionally is not identifiable externally. Care is taken to avoid tearing the dura, and a curette is used to locally expand the burr hole. Kerrison is then employed, and the goal is to get an approximate 1- to 2-cm width of bone, with the ultimate goal of the operation being to unlock the bones, not necessarily to create a wide gap. Once the burr hole and gap is created, it is locally expanded with more Kerrisons, and then we move superiorly toward the fontanelle. Here you can see through the endoscope the dura being dissected off of the undersurface of the bone with blunt dissection using a sucker and the endoscope. Bone-cutting scissors are then used to complete the craniectomy to midline. Once the two cuts have been made, the bone can be withdrawn. It is often connected to soft tissues, and a gentle twist of the bones will allow it to be excised in one large piece. The medial portion along the fontanelle should be visible.

After the medial portion has been removed, attention is turned inferiorly. Here you can see the sphenoid ridge internally. This is often the most problematic part of the operation, as the dura can be stuck in this region. If a dural tear is to be encountered, it is likely in this area, so we are quite careful. Once the dura has been dissected, we again use instruments including bone-cutting scissors and pituitary rongeurs to remove the bone all the way down to the lateral canthus. It is important to note that it is not technically viable or necessary to continue to follow the sphenoid, which continues anteriorly to the orbital roof. Once the sphenoid is crossed, the bony separation is angled more posteriorly to the squamosal suture. This will be seen in the postoperative CT scan. At the completion, we can see around the inferior temporal lobe, and one must be assured that the frontal and parietal bones are separated and mobile.

At this point, we have now turned attention to the patient's left side, but will not fully repeat the steps in this video. We separate the bones all the way to the fontanelle, palpating externally to be sure this has been achieved. Again, the medial bone is removed by twisting it where it is connected to the soft tissues, and you can see the feathery medial portion of the bone where the anterior fontanelle junction was.

Finally, attention is turned inferiorly on the left side. This endoscopic view shows the inferior extent. Once separation is complete, the bony edges are lined with Gelfoam, and we are quite rigorous about being sure all the bones are mobile. We have found that moist Gelfoam alone, without thrombin, is adequate for hemostatsis. We do not routinely cauterize the bone edges and use bone wax only selectively, but do routinely have our anesthesia team administer TXA for the syndromic cases. The incision is irrigated with bacitracin and closed in two layers using 4-0 Vicryls in the galea, followed by 4-0 Vicryl Rapide in the skin as a running suture. It is sterilely dressed. Our patients are generally not admitted to the intensive care unit unless there is an airway problem in the syndromic patients.

**6:55 CT of Immediate Postoperative Result**. In this slide, we show a CT scan which has the typical bony removal after bilateral endoscopic coronal suture removal in a patient with Apert syndrome

**7:05 Special Considerations for Syndromic Patients**. Compared to single-suture patients, there are increased risks of blood transfusion, hospital stay, and needing a secondary open operation at 29%. The indications for reoperation in the syndromic subset of our consecutive series of 500 patients was refusion of sutures and increased intracranial pressure in 72%, and inadequate cosmetic result in the fronto-orbital region in 28%, although all had nice correction of the turricephaly. Our subcategory numbers are small, but as of publication the reoperation rates are 18% of Apert, 50% of Muenke, 45% of Saethre Chotzen, and all of the Crouzon patients. Average time to reoperation was 1.9 years. Of note, when distractors are used, those patients need a second and third operation to remove distractors and then expand the front, so although 29% of patients needed a second operation, with posterior distractors 100% of patients need three operations.

**8:05 CT of Successful Posterior Result**. In this CT you can see the successful outcome in an Apert patient, where the coronal sutures remain patent and we had excellent correction of the turricephaly, and an excellent frontal expansion. No additional cranial surgery was needed.

**8:15 CT of Child Needing Reoperation**. In this Saethre-Chotzen patient the coronal sutures remain patent, but the sagittal and metopic sutures closed with a falloff in head circumference. A subsequent cranial expansion was necessary.

**8:30 References**^1–5^

## Acknowledgments

We would like to acknowledge James Koepfler, Medical Photography, Boston Children's Hospital.

## Author Contributions

Primary surgeon: Proctor. Assistant surgeon: Jeelani. Editing and drafting the video and abstract: Proctor. Critically revising the work: Proctor. Reviewed submitted version of the work: Proctor. Approved the final version of the work on behalf of both authors: Proctor. Supervision: Proctor.
